# Dynamics of Airborne Influenza A Viruses Indoors and Dependence on Humidity

**DOI:** 10.1371/journal.pone.0021481

**Published:** 2011-06-24

**Authors:** Wan Yang, Linsey C. Marr

**Affiliations:** Department of Civil and Environmental Engineering, Virginia Tech, Blacksburg, Virginia, United States of America; Erasmus Medical Center, The Netherlands

## Abstract

There is mounting evidence that the aerosol transmission route plays a significant role in the spread of influenza in temperate regions and that the efficiency of this route depends on humidity. Nevertheless, the precise mechanisms by which humidity might influence transmissibility via the aerosol route have not been elucidated. We hypothesize that airborne concentrations of infectious influenza A viruses (IAVs) vary with humidity through its influence on virus inactivation rate and respiratory droplet size. To gain insight into the mechanisms by which humidity might influence aerosol transmission, we modeled the size distribution and dynamics of IAVs emitted from a cough in typical residential and public settings over a relative humidity (RH) range of 10–90%. The model incorporates the size transformation of virus-containing droplets due to evaporation and then removal by gravitational settling, ventilation, and virus inactivation. The predicted concentration of infectious IAVs in air is 2.4 times higher at 10% RH than at 90% RH after 10 min in a residential setting, and this ratio grows over time. Settling is important for removal of large droplets containing large amounts of IAVs, while ventilation and inactivation are relatively more important for removal of IAVs associated with droplets <5 µm. The inactivation rate increases linearly with RH; at the highest RH, inactivation can remove up to 28% of IAVs in 10 min. Humidity is an important variable in aerosol transmission of IAVs because it both induces droplet size transformation and affects IAV inactivation rates. Our model advances a mechanistic understanding of the aerosol transmission route, and results complement recent studies on the relationship between humidity and influenza's seasonality. Maintaining a high indoor RH and ventilation rate may help reduce chances of IAV infection.

## Introduction

Influenza A has a clear seasonal pattern in temperate regions, yet the underlying cause for it remains controversial despite nearly a century of investigation. The literature identifies numerous factors that may influence influenza's seasonality: environmental conditions such as temperature, humidity, and ultraviolet radiation; immune function; school schedules; and human mobility patterns and contact rates [1]. Among these, the leading contenders are humidity and temperature [2], [3], [4], and in indoor environments, where people spend ∼90% of their time, humidity is the more variable factor. Particularly in the developed world where heating, ventilation, and air conditioning systems (HVAC) are the norm, indoor temperature tends to fall in a narrower range, and thus its influence is limited. Recent studies using a guinea pig experimental model [3], [4] indicate that low relative humidity (RH) favors aerosol transmission of influenza A viruses (IAVs), in which they are transmitted by small respiratory droplets expelled from infected hosts. Nevertheless, the precise mechanisms by which humidity might influence influenza viability and transmissibility via the aerosol route have not been elucidated.

Humidity may affect airborne IAV transmission via two important variables. The first is droplet size. When released from the respiratory tract (assumed to have 100% RH), droplets experience rapid evaporation and shrinkage upon encountering the unsaturated ambient atmosphere. The ultimate size of a droplet depends on ambient humidity, and size determines aerodynamic behavior and whether the droplet will settle to the ground quickly or remain suspended in the air long enough to possibly cause a secondary infection. Previous studies on evaporation of respiratory droplets usually used water or simple saline solutions (e.g., NaCl) to simulate respiratory fluid [5], [6]. However, respiratory fluid is a complicated combination of water, salts, and various organic compounds [7], [8] that affect the thermodynamics of evaporation, compared to pure water or saline solutions. The equilibrium droplet size is affected by surface curvature and solute effects, the combination of which is described by Köhler theory [9]. While the vapor pressure is enhanced over curved versus flat surfaces, it is reduced by the presence of solutes. These competing effects are magnified at smaller droplet diameters and determine the equilibrium size at a particular RH.

The second variable that is sensitive to humidity is IAV viability [2], [10], [11], [12], [13]. Hemmes et al. [2] linked influenza's seasonality to the seasonal oscillation of RH indoors, based on their experiment on death-rate variation versus RH. Shaman and Kohn [14], on the other hand, concluded that absolute humidity (AH) rather than RH modulates influenza seasonality through constraint on viability, but whether AH or RH is controlling is under debate. For our purposes, differentiating between the two is not possible because this work focuses on a narrow range of typical indoor temperatures. Finally, it is possible that the two variables—final droplet size and viability—are linked, if evaporation and subsequent concentration of solutes in respiratory droplets affects IAV viability in aerosols.

Elucidating the causes of influenza's seasonality will require improved comprehension of transmission mechanisms, especially the aerosol route. To advance a mechanistic understanding of the role of humidity in aerosol transmission, we model the change in size of respiratory droplets and IAV inactivation at RHs ranging from 10% to 90%. Based on these results, we further model the dynamics of droplets emitted from a cough in an indoor environment and illustrate the evolution of infectious IAV concentrations and size distributions, considering removal by gravitational settling, ventilation, and viral inactivation. We are thus able to determine the magnitude by which humidity affects airborne concentrations of infectious IAVs.

## Results

### Initial size distribution of droplets expelled from a cough

**Figure 1:**
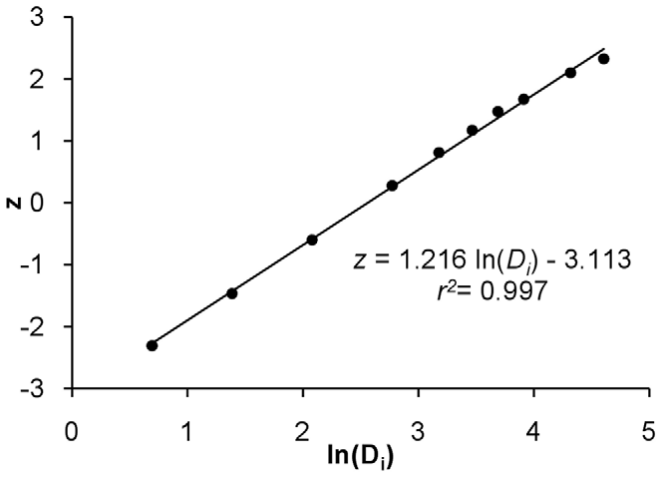
Log-probability plot of droplet size distribution from a cough, adapted from Duguid [**20**]. *D_i_* is the initial droplet size in µm, and *z* is the corresponding quantile of a normal distribution with the same cumulative probability.

We located seven papers that reported the size distribution of droplets expelled while coughing, sneezing, and speaking [6], [15], [16], [17], [18], [19], [20]. The droplet diameter geometric mean (GM), size range, and droplet number varied greatly among the different studies, as summarized in Table 1. Because ∼80% of patients with influenza manifest symptoms of coughing [21], we focus on droplets emitted from coughing to demonstrate the dynamics of airborne IAVs. Droplet diameters as small as 0.3 µm and as large as 2000 µm have been observed from coughing, and the GMs in the studies ranged from 0.25 µm to 96.6 µm, depending on the experimental methods used (Table 1). Four of the studies reported GMs in a much narrower range of 8.4 µm to 16.0 µm. We adopted Duguid's [20] results on the basis of reliability of the methods and care and thoroughness of the experimental design and analysis. Though the study dates to 1946, its results were similar to those of more contemporary work that use modern aerosol characterization equipment [6], [17]. Similar to Nicas et al. [22], we found that droplets between 1–100 µm from coughing follow a log-normal size distribution. Eq. 1 describes the relationship:
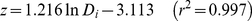
(1)where *D_i_* is the initial droplet diameter in µm, and *z* is the corresponding quantile of a normal distribution with the same cumulative probability. According to this relationship, the size distribution of droplets emitted from coughing has a GM of 12.9 µm and a geometric standard deviation (GSD) of 2.3 (Figure 1).

**Table 1:** Prior studies of respiratory droplet size distributions.

Activity	Droplet size (µm)		Droplet number	Experimental conditions	Measurement methods	Adjustment for evaporation^b^	Reference
	GM (GSD)^a^	Range					
Cough	12.1 (2.6)^c^	1–2000	5000	NA	Microscope	Factor of 4	[20]
	16.0 (5.8)^d^	1→1471	466	NA	Bone paper and 0.45-µm filter	As measured	[15]
	0.5 (1.7)^e^	<0.6–2.5	420	24°C, 45%RH;35°C, 23%RH	Optical particle counter (OPC), electron microscope	As measured	[16], [22]
	8.4 (2.2)	NA	NA	95% RH	Aerodynamic particle sizer (APS), scanning mobility particle sizer, OPC	As measured, assumed to be the original size	[17]
	13.5^f^	2–1000	NA	24.9°C, 73.9% RH	Interferometric Mie imaging (>2 µm), particle image velocimetry	As measured	[6]
	1.8^g^	0.3–20	NA	27°C, 59.4% RH	Expiratory droplet investigation system, APS	As measured	[18]
	96.6 (2.4)^h^	0–1500	42	28°C, 70% RH	Microscope, aerosol spectrometer	Factor of 3	[19]
Sneeze	8.2 (2.3)^c^	1–2000	1×10^6^	NA	Microscope	Factor of 4	[20]
Speak^i^	11.9 (2.8)^c^	1–1000	252	NA	Microscope	Factor of 4	[20]
	16^f^	2–1000	NA	24.9°C, 73.9% RH	Interferometric Mie imaging (>2 µm), particle image velocimetry	As measured	[6]
	62.1 (1.8)^h^	0–1000	253	28°C, 70% RH	Microscope, aerosol spectrometer	Factor of 3	[19]

a Geometric mean (GM) and geometric standard deviation (GSD) calculated by methods presented in [51] or cited as reported in the original papers.

b Whether droplet sizes were adjusted upward to account for evaporation or were reported as measured.

c Calculated from data in Table 3 in [20].

d Calculated from data in Table 1 in [15]; droplet diameter upper end assumed to be 2000 µm.

e Calculated from data in Table IV in [22].

f No data on GSD reported.

g Reported modal diameter.

h Calculated from data in Table 2 in [19], only results from experiments without food dye were used.

i Counting aloud from 1 to 100.

### Respiratory droplet size transformation

Equilibrium droplet size is attained nearly instantaneously upon release. A 20-µm droplet shrinks to one-half of its original diameter in less than half a second at 20°C [22]. Table 2 shows the equilibrium, or final, diameters (*D_eq_*) of droplets with *D_i_* of 0.1, 1, and 10 µm at 10–90% RH. These were calculated based on a model of droplet transformation that assumes separate solutes and volume additivity (SS-VA) [23]. Due to the Kelvin effect, evaporation of smaller droplets is enhanced, and the equilibrium diameters are smaller. The ratio *D_eq_*/*D_i_* is 0.490 at 90% RH for a respiratory droplet with *D_i_* = 0.1 µm, versus 0.516 under the same conditions for a larger one with *D_i_* = 10 µm. However, the Kelvin effect is negligible for droplets with *D_i_*>0.1 µm, and *D_eq_*/*D_i_* is independent of RH for droplets larger than 1 µm.

**Table 2:** Respiratory droplet size transformation.

RH	Model-based *D_eq_*/*D_i_* ratios^a^			Experimentally derived *D_eq_*/*D_i_* ratios^b^	Diff.^c^
	*D_i_* = 0.1 µm	*D_i_* = 1 µm	*D_i_* = 10 µm		
10%	0.401	0.402	0.402	0.391	2.61%
20%	0.407	0.407	0.407	0.395	3.06%
30%	0.412	0.412	0.412	0.398	3.42%
40%	0.416	0.417	0.417	0.401	3.98%
50%	0.422	0.423	0.424	0.427	−0.90%
60%	0.429	0.431	0.432	0.437	−1.19%
70%	0.439	0.443	0.444	0.449	−1.20%
80%	0.456	0.464	0.465	0.464	0.02%
90%	0.490	0.513	0.516	0.502	2.63%

a Calculated according to the SS-VA model of Mikhailov et al. [23].

b Calculated based on volume additivity using experimental data from Tang et al. [25] and Bagger et al. [26].

c Difference between modeled and experimental *D_eq_*/*D_i_* ratios for *D_i_* = 10 µm.

Efflorescence or crystallization of NaCl, a major component of respiratory droplets, due to loss of water is expected to occur between 40–50% RH [24]. According to our results, between 10–40% RH, *D_eq_*/*D_i_* varies by only 3.7% (0.402–0.417); in comparison, between 50–90% RH, the ratio varies by 21.7% (0.424–0.516). Respiratory droplets lose almost all their water at low RHs. For comparison, we also calculated the *D_eq_*/*D_i_* ratios based on volume additivity using experimental data on dehydration of droplets containing NaCl [25] and on hydration of those containing a glycoprotein [26]. Differences between modeled and experimental results are less than 4% (Table 2).

### Inactivation of airborne IAVs

**Figure 2:**
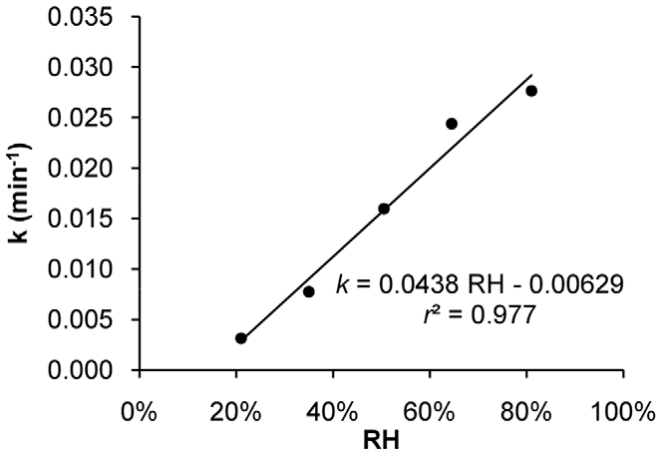
IAV inactivation rate versus RH. IAV inactivation rates (*k*) for each RH over 1 h were calculated based on experimental data adapted from Harper [12].

The viability of IAVs decreases over time and is affected by environmental variables such as temperature, humidity, and UV radiation [12], [27]. The inactivation rate (*k*), derived from experimental data on airborne IAVs [12], is linearly correlated with RH (Figure 2), following the relationship
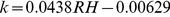
(2)with an *r* ^2^ of 0.977 and *p*-values for the model, intercept, and slope of 0.0015, 0.059, and 0.0015, respectively.

### Evolution of infectious IAV distribution after a cough

The well-mixed indoor air model we developed for infectious IAVs accounts for removal by gravitational settling, ventilation, and viral inactivation. The concentration of infectious IAVs associated with droplets of a specific diameter *D_eq_* in a room at time *t* is
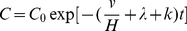
(3)where *C_0_* is the initial concentration of infectious IAVs associated with droplets of size *D_eq_* in the room, *v* is the settling velocity, *H* is the height of the room, *λ* is the air exchange rate (AER) and assumes no recirculation, and *k* is the inactivation rate. The inactivation rate *k* depends on RH, according to Eq. 2, and the settling velocity *v* depends on *D_eq_*, which also depends on RH. Eq. 3 can be integrated over all droplet sizes to obtain the total concentration of infectious IAVs in a room. We calculate results for AERs of 1 air change per hour (ACH) and 10 ACH, typical of residential and public settings, respectively [28], [29]. For simplicity, we assumed that room heights are the same in residences and public settings such as offices, classrooms, and hospitals, where people aggregate and thus have a higher risk of infection. The equation is applicable for a single rectangular room, does not depend on the volume of the room, and does not account for air exchange between multiple rooms.

**Figure 3:**
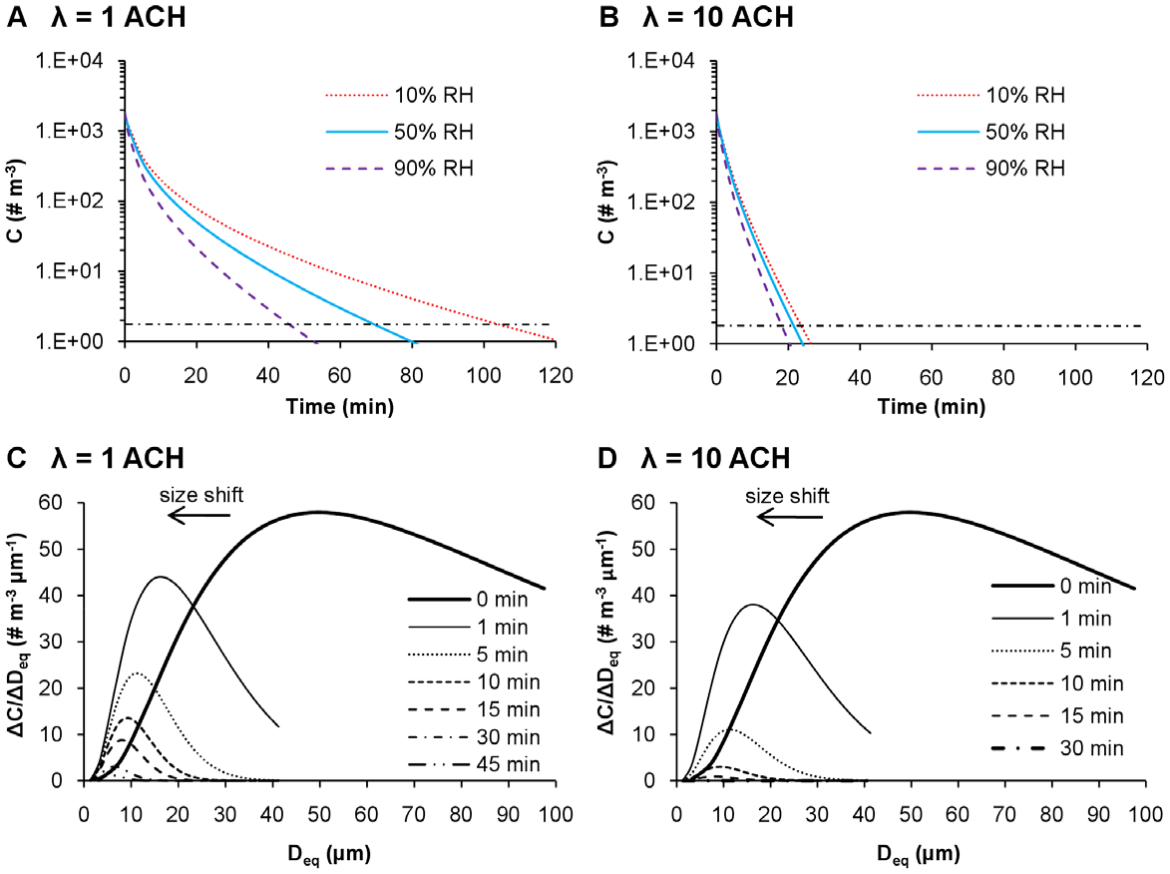
Evolution of infectious airborne IAV concentrations and size distributions. Time series of airborne, infectious IAV concentrations following a cough into residential (A) and public (B) settings at 10–90% RH. The horizontal dashed line indicates 99.9% removal. Evolution over time of airborne, infectious IAV size distribution following a cough into residential (C) and public (D) settings at 50% RH.

Figure 3 shows the evolution of IAV concentrations in time in terms of both the total number of infectious viruses (Figure 3A and 3B) and size distribution (Figure 3C and 3D). Only emitted droplets with *D_i_*≤100 µm are considered, as larger ones will be removed by gravitational settling within seconds. Following a single cough in a well-mixed room, the concentration of infectious IAVs is initially 1.8×10^3^ # m^−3^ under the assumptions of this study. Figures 3A and 3B show that the total number of infectious IAVs falls rapidly with time and that the loss is greater at higher RH and in public versus residential settings. If one infected person is continuously shedding viruses by coughing 15 times per hour [30], then the concentration of IAVs will be ∼2×10^3^ # m^−3^ in a public setting. This concentration is similar in magnitude to those measured in hospitals, medical clinics, day care facilities, and airplanes [31], [32], [33]. Under conditions of higher RH, removal by settling is more effective because droplets shrink less, and inactivation is more rapid. Removal of 99.9% of the IAVs emitted requires much greater time in a residential versus public setting, indicating that ventilation is an important removal mechanism and that airborne IAVs can persist for longer times in settings with lower AERs.

Figure 3C and 3D show that, at 50% RH, emitted droplets shrink to about half of their original diameters due to evaporation. Evaporation happens almost instantaneously [22], so while the initial size distribution at 0 min extends out to 100 µm, all subsequent ones end at 42 µm (*D_eq_*/*D_i_* = 0.42). This process greatly increases the fraction of IAVs that are associated with smaller droplets, since the virus concentration within a droplet increases by a factor of 8 (2^3^) as its diameter shrinks by half. For instance, compared to the initial droplets emitted at 0 min, which quickly reach their equilibrium diameters, the number of IAVs associated with equilibrium droplet sizes smaller than 25 µm increases by a factor of 5.2 at 50% RH. Due to more rapid settling, IAVs associated with larger droplets are lost faster than are those associated with smaller ones. Consequently, IAVs associated with smaller droplets become more dominant over time, as indicated by the shifting of the peak of Δ*C*/Δ*D_eq_* to the left in Figure 3C and 3D. The diameter of droplets containing the most IAVs (i.e., the mode of the distribution) shifts from ∼50 µm upon release to ∼16 µm at 1 min, ∼10 µm at 10 min, and ∼5 µm at 60 min in residential settings; a similar trend is shown in Figure 3D for public settings.

### Humidity dependency and removal mechanisms

**Figure 4:**
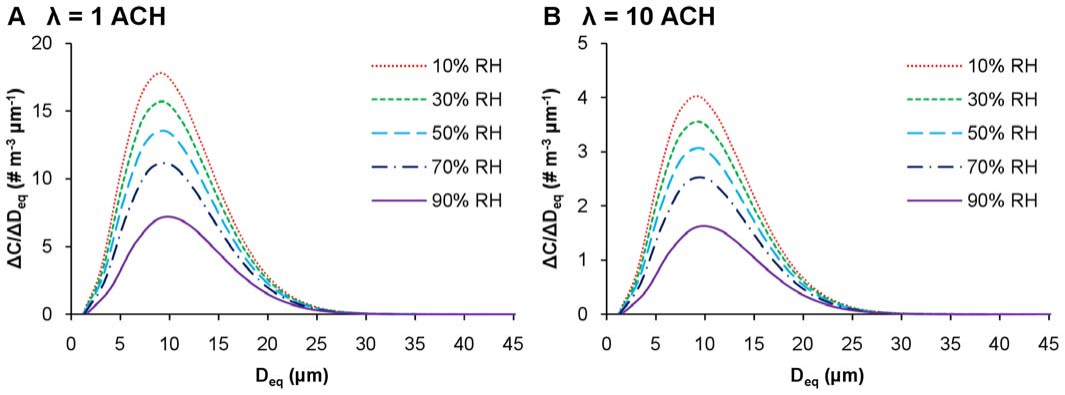
IAV size distributions. Infectious IAV size distributions at various RHs in residential (A) and public (B) settings with a volume of 50 m^3^ and a height of 2.5 m, 10 min after a cough.

Figure 4 shows the effect of RH on size distributions of infectious IAVs, 10 min after a cough in residential and public settings. For both cases, IAV concentrations decrease with increasing RH across all sizes, but the modes of the distributions remain around 9–10 µm. The total IAV concentration (i.e., the area under each curve) decreases with increased RH. As a result, it takes twice as long to remove 99.9% of IAVs emitted at 10% RH than that at 90% RH in residential settings (>100 min at 10% RH versus <50 min at 90% RH, as shown in Figure 3A).

Humidity affects both settling, because of its dependence on size transformation, and inactivation of IAVs. The relative importance of these two effects can be illustrated by comparing the ratios of virus concentrations at 10% RH versus those at 90% RH at varying times. The ratios increase approximately exponentially with time: 2.4 at 10 min, 5.4 at 30 min, and 16.1 at 60 min. If only inactivation were considered, these factors would instead be 1.4, 2.7, and 7.3, respectively; and if only settling were considered, the corresponding factors would be 1.7, 2.0, and 2.2, respectively. These ratios are independent of the ventilation rate. The much narrower range of factors for settling than for inactivation (i.e., 1.7–2.2 versus 1.4–7.3) indicates that RH has a greater impact on inactivation, especially over long periods (>30 min).

**Figure 5:**
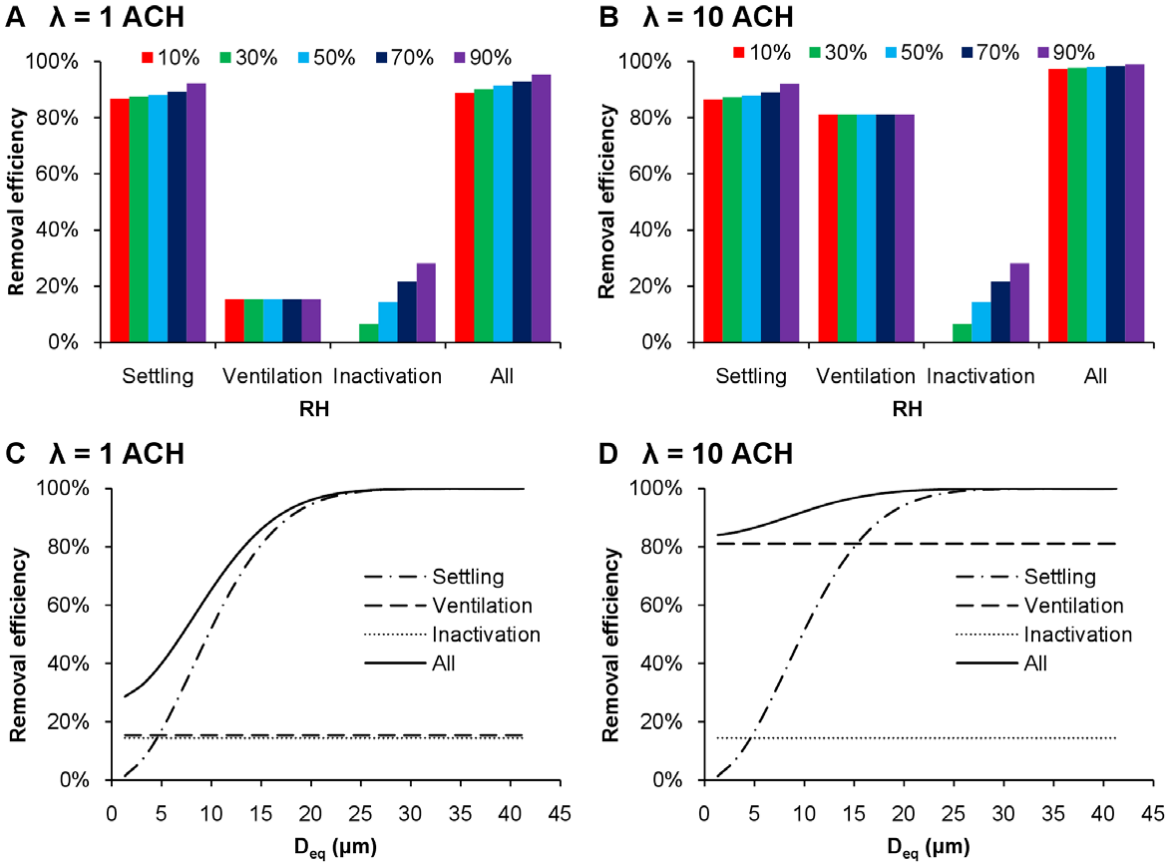
IAV removal mechanisms. Infectious IAV removal efficiencies due to settling, ventilation, and inactivation in residential (A) and public (B) settings at different RHs. Removal efficiency of settling, ventilation, and inactivation as a function of droplet size in residential (C) and public (D) settings at 50% RH. Removal efficiencies are shown for each mechanism independently and do not sum to 100% because in actuality, more than one mechanism may act on the same virus/droplet.

Figure 5 shows the effectiveness of each removal mechanism—settling, ventilation, and inactivation—independently as a function of RH (Figure 5A and 5B) and droplet size (Figure 5C and 5D), 10 min following a cough. Figure 5A shows that gravitational settling is the dominant removal mechanism in residential settings. Settling alone removes over 80% of airborne IAVs within 10 min, and its removal efficiency increases slightly with RH, from 87% to 92% across the range of RHs. In contrast, ventilation only removes 15% of total IAVs, regardless of RH. Removal efficiency by inactivation increases with RH, accounting for up to 28% at the highest RH. Figure 5B shows that ventilation and gravitational settling are both important in removing airborne IAVs from public settings with higher AERs. At an AER of 10 ACH with no recirculation, ventilation removes 81% of airborne IAVs. Settling and inactivation are independent of ventilation rate and remove the same amounts of IAVs as in residential settings.

Removal efficiencies for IAVs vary as a function of droplet size for settling but not ventilation or inactivation. Figure 5C and 5D show removal efficiency versus equilibrium droplet diameter at 50% RH, 10 min following a cough, for each mechanism individually and all three together. Because settling velocity scales with diameter squared, removal efficiencies due to gravitational settling range from only 0.7% for droplets with *D_eq_* = 1 µm to 51.2% for those with *D_eq_* = 10 µm to >98.8% for those with *D_eq_*>25 µm. Ventilation is equally effective for all sizes, with removal efficiencies of 15% in residential settings (Figure 5C) and 81% in public settings (Figure 5D), depending on the AER.

Overall, gravitational settling is the main removal mechanism in both residential and public settings (Figure 5A and 5B). It removes a disproportionately large fraction of IAVs because it favors larger droplets, which contain far more IAVs, as their numbers are proportional to the initial droplet volume, or *D_i_* ^3^. However, settling is ineffective at removing droplets <5 µm, as shown in Figure 5C and 5D. Ventilation is important in public settings and particularly so for removal of smaller droplets (<5 µm) for which settling is inefficient. It accounts for ∼50% of total removal of IAVs associated with droplets <5 µm in residential settings (Figure 5C) and ∼80% in public settings (Figure 5D). Inactivation increases with RH and is maximal at 28% at 90% RH, 10 min following a cough (Figure 5A and 5B). Although the removal efficiency by inactivation is relatively low, it is important when removal by ventilation and settling are both minor. For instance, in residential settings (Figure 5C), inactivation accounts for ∼50% of total removal of IAVs associated with droplets <5 µm.

## Discussion

### Removal of infectious IAVs

Higher RH favors removal of infectious IAVs. Since larger droplets have greater settling velocities, higher RHs, at which *D_eq_*/*D_i_* is larger, thereby will accelerate the removal rate. Additionally, the inactivation rate of IAVs increases with increasing RH (Eq. 2). According to our model, the concentration of airborne IAVs resulting from a cough would be reduced by 10% if the RH increases from 35%, the mean indoor RH in heating season [30], to 50%, 10 min following the cough, and by 40% after 1 h in residential settings. These estimates agree in magnitude with those reported by Myatt et al. [30], whose model suggests that influenza virus survival decreases by 17.5–31.6% when indoor RH increases by 11–19% over 15 h. Hence, maintaining a reasonably high indoor RH (e.g., 50%) may accelerate the removal of infectious IAVs and help prevent or reduce influenza infection.

The relative importance of the two mechanisms—droplet size transformation and inactivation—as a function of humidity is of interest. Shaman and Kohn [14] concluded that AH modulates influenza transmission by influencing the virus' survival rate, rather than by enhancing production of airborne droplet nuclei in low humidity conditions. We found that respiratory droplets would shrink to one-half of their original diameters at 90% RH, and to around two-fifths at 10% RH. It thus appears that changes in droplet size are dramatic at unsaturated RHs and that variations due to differences in RH are relatively trivial. Our analysis shows that removal by inactivation is more variable with RH than is removal by settling. This may explain why Shaman and Kohn [14] could find a statistically significant relationship between AH and influenza survival but not transmission. However, this does not suggest that droplet shrinkage in response to unsaturated RHs is not important for influenza transmission, only that it is not as obvious as the induced change in viability.

We have demonstrated the relative importance of the three removal mechanisms. Settling can remove over 80% of droplets emitted from a cough within 10 min; however, it is effective only for larger droplets and allows the smaller ones (<5 µm) to remain suspended. In contrast, ventilation is able to remove all droplets regardless of size simply by air exchange. Therefore, higher AERs will facilitate the elimination of virus-containing droplets from indoor environments, especially to compensate for the inefficacy of settling in removing the small ones. This observation also justifies the requirement to maintain a high AER in public places (e.g., 12 ACH in hospital waiting areas [34]). Removal efficiencies due to virus inactivation are relatively small (i.e., 0–28% in 10 min, if only inactivation were considered). However these estimates are based on experimental data reported by Harper [12], which indicated lower inactivation rates of 0.0031–0.028 min^−1^ at 20–81% RH, compared to 0.0073±0.0031 min^−1^ at 15–40% RH and 0.091±0.024 min^−1^ at 50–90% RH, as reported by Hemmes [2]. If estimated Hemmes' data [2], the corresponding removal efficiencies would be larger: 7.0% at 15–40% RH and 59.8% at 50–90% RH. Virus inactivation may thereby play a more significant role depending on the actual inactivation rate.

### IAV viability, seasonality, and humidity dependency

Experimental and/or theoretical models have been constructed to predict the viability of airborne IAVs as a function of humidity [14], [35], but a widely accepted mechanistic explanation for the relationship is still lacking. Studies on the effect of humidity agree that IAVs survive better at lower RHs. However, Hemmes [2], [11] and Harper [12] found higher inactivation rates at both medium and high RHs, in contrast to Shechmeister [10] and Schaffer et al. [13], who found higher inactivation rates at medium but not high RHs. This disparity may stem from the different compositions of media used in each experiment. All media contained salts (approximately 0.5–3%); however, those used in the former two experiments contained far more proteins than did those in the latter two. High concentrations of salts are found to be detrimental to avian IAVs [36]. As water in the droplets evaporates, solute concentrations increase and may consequently accelerate IAV inactivation. However, an NaCl droplet can suddenly lose all of its water and crystallize at the point of efflorescence (45–48% RH) [25], thus eliminating the negative effect of dissolved salts at low RHs. This effect is perceivable in Table 2, which shows that *D_eq_*/*D_i_* varies little when RH≤40%. The combination of increasing salt concentrations followed by efflorescence as RH decreases may explain the trend observed by Shechmeister [10] and Schaffer et al. [13].

Additionally, a study on aerosol transmission between guinea pigs [3] indicated that transmission was inversely related to RH at 5°C, although experiments at 20°C showed a lower transmission rate at 50% RH than at 65% RH. As 0% transmission was observed at 80% RH, the inconsistent result at 50% or 65% RH may be due to the stochastic nature of infection. If higher transmission rates are due to higher viabilities, at least in part, these results appear to agree with the trend reported by Hemmes [2], [11] and Harper [12]. Given the similar constitution of droplets emitted from infected human and guinea pigs (i.e., salts plus proteins), it seems reasonable to believe that IAVs associated with droplets expelled from humans will be subject to higher inactivation at higher RHs.

The relationship between IAV viability and RH may be due to interactions among components of respiratory droplets (i.e., glycoproteins, salts, and water) and the virus that are sensitive to concentration, which depends on the extent of evaporation, which depends in turn on ambient humidity. Proteins may complicate the effect of salt ions on IAVs by interacting with the salt ions and counteracting their adverse effects. Studies have shown that IAVs remain infectious much longer in the presence of respiratory mucus [37], [38]. Investigation into such interactions and the possible complexes formed in respiratory droplets in response to humidity variation at a molecular level is needed.

We speculate that the seasonality of influenza with its wintertime peak in temperate regions is stimulated by more vigorous evaporation of droplets at low RHs leading to higher suspended concentrations of IAVs, combined with the sensitivity of aerosolized IAVs' viability to RH. When RH is <90%, droplets shrink approximately in half, leaving associated IAVs that can remain suspended long enough to cause secondary infections. Our recent measurements of size-resolved airborne IAV concentrations support this assertion: 64% of the IAV genomes detected in a daycare center, a health center, and airplanes were associated with fine particles <2.5 µm (15% in the 0.25–0.5 µm fraction, 10% in the 0.5–1.0 µm fraction, and 28% in the 1.0–2.5 µm fraction) [33]. These particles can remain suspended for hours to days. Because of the many factors involved in infection, it is still not clear which size of droplets is most likely to transmit influenza, nor is it clear which region in human airways is most susceptible to influenza infection. However, if we simply consider deposition efficiency in human airways, the droplet size with the highest deposition efficiency (∼95%) in all regions of the airways combined is ∼5 µm. For such droplets, deposition efficiency is ∼10% in the tracheobrochial and alveolar regions; the majority of the droplets deposit in the nasopharyngeal region. The droplet size with the highest deposition efficiency (∼17%) in the tracheobronchial and alveolar regions is ∼2.5 µm [39]. Thus these smaller droplets have greater potential both to remain suspended and to deposit deeper into human airways.

At extremely high RHs, for example, close to 100% in tropical regions during the rainy season, the droplets do not shrink as much (*D_eq_*/*D_i_* = 0.927 at 99% RH, and 0.755 at 98% RH, according to our calculations). Droplets thus settle more quickly, rendering the aerosol route relatively less important. However, due to less evaporation, salts and glycoproteins remain at concentrations closer to those found in the respiratory tract, and these concentrations are not detrimental to the virus. As suggested by Lowen et al. [40], [41], other transmission routes (e.g., contact) may dominate in the tropics. They also proposed that the airborne route's sensitivity to RH and temperature contributes to seasonality in temperate regions while the contact route's insensitivity to the two variables contributes to year-round influenza in tropical regions. Our analysis supports this hypothesis.

### Model limitations

There are several limitations of our model. First, although the model used to predict equilibrium droplet sizes has been confirmed with experiments using NaCl-bovine serum albumin (BSA) particles [23], further verification with actual respiratory fluid is needed due to its complex composition. Furthermore, the composition of respiratory fluid depends on the emission site (nose or mouth) and source (upper or lower respiratory tract), as well as the stage of infection. Inflamed airways secrete larger amounts of mucus which consequently increase the dry mass of respiratory fluid [42]. Therefore, the equilibrium size of emitted droplets may be larger than presented here based on composition under healthy conditions. On the other hand, saliva has much lower concentrations of salts and glycoproteins [43], [44], due to dilution by which droplets emitted from coughing may have lower dry mass.

Second, the model is based on limited data obtained from laboratory experiments. Not only are Harper [12] and other studies of IAV viability in aerosolized droplets [10], [11], [13] decades old, but none investigated inactivation rates as a function of droplet size. More accurate measurements concerning the influence of respiratory droplet size on IAV viability are needed to better predict the fate of airborne IAVs.

Third, we calculated the IAV concentration based on a well-mixed room model with no recirculation. This model assumes that droplets are instantaneously, continuously, and evenly distributed throughout the room. However, according to Lai and Cheng [45], it takes at least 270 s for 10-µm droplets to mix thoroughly at 5 ACH. It may take even longer for the system to become well-mixed at lower AERs. More accurate calculations may be achieved by the use of computational fluid dynamics. Additionally, if recirculation accounts for a large fraction of the AER and if viruses are not removed in the HVAC system, then ventilation will play a relatively smaller role in virus removal compared to settling and inactivation.

Finally, this research demonstrates the evolution of IAV concentrations induced by a cough but not other activities, such as normal respiration, talking, and sneezing. Fabian et al. [46] determined that IAV RNA (i.e., potentially infectious IAVs) was emitted in exhaled breath from infected patients at a rate of <3.2 to 20 RNA particles min^−1^. These droplets were smaller than those associated with a cough; over 87% of them were <1 µm in diameter. Therefore, for IAVs exhaled during normal breathing, because of the smaller droplet size, airborne IAV concentrations would be lower and removal would rely more on ventilation and inactivation than gravitational settling. IAVs may also be expelled during talking, although to our knowledge, no detailed experimental data on this phenomenon are currently available in the literature. The droplet size distribution for talking is similar to that for coughing (Table 1). Therefore, model results are expected to be similar for IAVs generated by talking in terms of removal efficiencies by different mechanisms. Sneezing is a less common clinical manifestation of influenza than is coughing, which is manifested in ∼80% of patients [21], [46], [47], [48]. The main difference between sneezing and coughing is that the former generates far more droplets, especially smaller ones. Thus, IAV concentrations would be higher initially, and ventilation would play a larger role in removal.

## Methods

### Equations for generating the initial respiratory droplet size distributions

From data on the size distribution of droplets expelled in a cough [20], we considered counts of droplets with *D_i_*≤100 µm. The standard normal distribution *z*-value with the same cumulative probability as that for droplets with a diameter *D_i_* was computed by the NORMSINV function in Excel 2007. The equation of the least-squares linear regression between z and ln*D_i_* is shown in Eq. 1 and Figure 1.

The initial size distribution of droplets (≤100 µm) from coughing was then generated by the NORMSDIST function in Excel 2007:
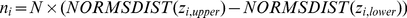
(4)where *n_i_* is the droplet count in the *i*th size bin (5-µm step in this study), *N* is the total number of droplets ≤100 µm (i.e., 4775 according to Duguid [20]), and *z_i, upper_* and *z_i, lower_* are the upper and lower *z* values of the *i*th size bin.

### Model for calculating equilibrium respiratory droplet size

The equilibrium droplet sizes resulting from evaporation were estimated based on Köhler theory taking into account the two major constituents of respiratory fluid: inorganic salts and glycoproteins. Effros et al. [7] determined concentrations (mean ± standard error) of the major electrolytes to be, respectively, 91±8 (Na), 60±11 (K), and 102±17 (Cl) mM, of glycoproteins to be 76.3±18.2 g L^−1^, and of lactate to be 44±17 mM. We thereby assume respiratory fluid contains 150 mM (8.8 g L^−1^) NaCl to represent the inorganic components and 76 g L^−1^ of total proteins (TP) to approximate the organic components, as done by Nicas et al. [22].

The SS-VA model derived by Mikhailov et al. [23] is based on the physiochemical properties (practical osmotic coefficients, molecular weights, and densities of the component solutes, etc.) of the droplet and the Kelvin effect. Their modeling results for particles with 90% BSA (dry mass fraction) fitted well with experimental data for dehydration of mixed NaCl-BSA particles. Given the similar composition of respiratory fluid (89.6% TP in dry mass) to their NaCl-BSA particles, we applied their SS-VA model to compute the equilibrium size for respiratory droplets.

The SS-VA model predicts the equilibrium RH with a specific droplet diameter (*D_eq_*) to be:
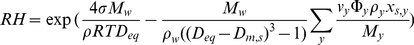
(5)where, *σ* is the surface tension (approximated by that of water as done in Mikhailov et al. [23], i.e., 0.072 N m^−1^); *M* is the molar mass, the subscripts *w* and *y* refer to water and component *y* (either NaCl or TP), respectively, and *M_w_* = 18 g mol^−1^, *M_NaC_*_l_ = 58.4 g mol^−1^, *M_TP_*≈*M_BSA_* = 66.5×10^3^ g mol^−1^; *ρ*, *ρ_w_*, *ρ_y_* are the densities of the entire droplet, water, and component *y* (*ρ_NaCl_* = 2165 kg m^−3^, *ρ_TP_*≈ρ*_BSA_* = 1362 kg m^−3^), respectively; *R* is the ideal gas constant; *T* is the absolute temperature (298 K in this study); *D_eq_* is the equilibrium diameter of a droplet residue at a given RH; *D_m,s_* is the mass equivalent diameter of a particle consisting of the dry solutes; *υ_y_* is the stoichiometric dissociation number of component *y*, *υ_NaC_*_l_ = 2, and *υ_TP_* = *υ_BSA_* = 1; *Φ_y_* is the molal or practical osmotic coefficient of component *y* describing the non-ideality of the solution; and *x_s,y_* is the mass fraction of component *y* (*x_NaCl_* = 0.104 and *x_TP_* = 0.896 in this study). Given *D_i_*, the mass equivalent diameter, *D_m,s_*, can be calculated and used as an input to further calculate *D_eq_* with its equilibrium RH.

### Virus inactivation rate

Harper [12] performed a detailed study on the viability of airborne IAVs over a wide range of both RH and temperature. In the experiment, droplets containing IAVs were generated with an atomizer and stored in a drum turning at 3 rpm, and results were corrected for physical loss by settling and other deposition mechanisms. We used his viability data at 20–24.5°C, typical indoor temperatures, at RHs ranging from 20% to 81% to calculate inactivation rates as a function of RH (Table 3). Because the residence time of air indoors is typically 1–2 h at most, we considered viability data from the first 1 h of the experiment only.

**Table 3:** Inactivation of airborne IAVs at 20–24.5°C over 1 h.

T (min)	Viability (*S_t_*) at different RHs^a^				
	20–22%	34–36%	50–51%	64–65%	81%
0.017 (1 s)	0.75	0.86	0.84	0.77	0.67
5	0.77	0.93	0.62	0.45	0.55
30	0.65	0.58	0.49	0.29	0.22
60	0.64	0.59	0.29	0.15	0.13

a Experimental data from Harper [12].

b Average RH.

c *k* = slope of ln(*S_t_*) versus *t*.

d Intercept of the plot of ln(*S_t_*) versus *t*.

e *r* ^2^ of the plot of ln(*S_t_*) versus *t*.

We quantified viability by assuming that airborne IAVs undergo first-order inactivation upon emission, such that,
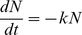
(6)where *N* is the number of IAVs emitted, *t* is time, and the inactivation rate (*k*) is
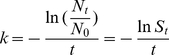
(7)where *N_0_* and *N_t_* are the numbers of IAVs at *t* = 0 and time *t*, and *S_t_* is the survival rate, or viability (%) at time *t*. Accordingly, we computed *k* for each RH from the *S_t_* data reported by Harper [12] using the SLOPE function in Excel 2007 (Table 3). The equation of the least-squares linear regression between *k* and *RH* is shown in Eq. 2.

### Concentration of infectious IAVs indoors

The model for estimating the concentration of infectious IAVs assumes that they are emitted from a cough and instantaneously well-mixed within the whole indoor space such that IAV concentrations in the room and outlet air are the same. The IAVs are subjected to removal by ventilation, inactivation, and gravitational settling. Droplet size transformation is assumed complete at time zero, and *D_eq_* was used for the calculation. Assuming the inlet air contains no IAVs, the change of IAV concentration with time is modeled as:
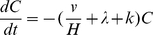
(8)where *C* is the infectious IAV concentration in the room and outflow (# m^−3^); *v* is the gravitational settling velocity calculated by Stokes law based on *D_eq_*; *H* is assumed to be 2.5 m in this study; λ is the air exchange rate assuming no recirculation; and *k* is the inactivation rate given by Eq. 2.

At time zero, IAVs are released from a cough, and the initial concentration of IAVs associated with droplets in the *i*th size bin, *C_i,0_* (# m ^−3^) is
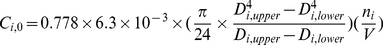
(9)where 0.778 is the initial survival rate (i.e., the average survival rate at 1 s according to the results of Harper [12]); 6.3×10^−3^ is the IAV concentration in respiratory fluid (# µm^−3^) obtained by assuming that the respiratory fluid contains the same concentration of IAV as in nasal washes of infected persons (6.3±0.3×10^6^ median tissue culture infectious dose (TCID_50_) mL^−1^ [49]) and that 1 TCID_50_ equals 1000 virus particles [50]; the term in the first set of parentheses is the mean droplet volume for the bin; *D_i,upper_* and *D_i,lower_* are, respectively, the upper and lower diameters of the *i*th size bin (µm); *n_i_* is the droplet count for the *i*th size bin given in Eq. 4, and *V* is the room volume (assumed to be 50 m^3^). The solution for *C* is given in Eq. 3.

### Removal efficiency of settling, ventilation, and inactivation

Removal efficiency in this study refers to the percentage of IAVs removed by a certain mechanism (i.e., settling, ventilation, inactivation, or a combination of these three) at a given time and RH. In Figure 5, removal efficiencies of settling (*E_settling_*), ventilation (*E_vent_*), and inactivation (*E_inactivation_*), and total removal efficiency (*E_total_*) are calculated by Eq. 10–13:
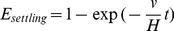
(10)

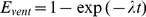
(11)

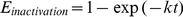
(12)

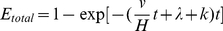
(13)

